# The Role of Vitamin D in Inflammatory Bowel Disease

**DOI:** 10.3390/healthcare3020338

**Published:** 2015-05-27

**Authors:** Aaron S. Bancil, Andrew Poullis

**Affiliations:** 1 Epsom Hospital, Dorking Road, Epsom, Surrey, KT18 7EG, UK; 2 St George’s Hospital, Blackshaw Road, London, SW17 0QT, UK; E-Mail: andrew.poullis@stgeorges.nhs.uk

**Keywords:** Inflammatory Bowel Disease, vitamin D, Crohn’s Disease, Ulcerative Colitis

## Abstract

Vitamin D is known to be vital in maintaining bone health, mineralisation and for fracture prevention. It has also been implicated in a number of autoimmune diseases and has therefore been studied for its potential role in Inflammatory Bowel Disease (IBD). This review looks at the current literature on the role of vitamin D and its potential role as an immunomodulator, disease modifier and bone health in IBD patients. There is substantial supporting evidence of an important role from epidemiological, genetic and immunological studies, but there is also conflicting evidence and nothing proving to be definitive from clinical studies. There are also a number of confounders with IBD patients, as their lifestyles and medications may affect vitamin D levels. Murine studies have added vast amounts to our knowledge of vitamin D and its antimicrobial role, as well as its effect on immune cell proliferation other inflammatory molecules, such as Tumour Necrosis Factor-α (TNFα). It is clear that larger trials investigating the effects of oral supplementation of vitamin D in IBD patients are necessary.

## 1. Introduction

Inflammatory Bowel Disease (IBD) is a term that encompasses mainly Crohn’s Disease (CD) and Ulcerative Colitis (UC). The pathophysiology is not fully understood but is thought to be caused by a complex interplay between gut microbiota, dysregulation of the host’s immune system, genetic susceptibility and environmental factors [1]. A number of other factors have been implicated for their role in IBD pathophysiology. Research has looked into vitamin D’s potential involvement with disease pathogenesis, severity and perhaps treatment.

It is known that vitamin D is vital in the maintenance of bone strength, mineralisation and fracture prevention [2]. Vitamin D’s physiological importance has also been implicated in a number of inflammatory diseases, mainly asthma, atherosclerosis and autoimmune disease [3]. Thus, vitamin D deficient IBD patients are at an increased risk of fractures and low bone density, especially if they have longstanding disease, or have long courses of steroids [4].

Vitamin D deficiency is common amongst IBD patients, yet there is increasing evidence for the vitamin’s role in disease activity [5]. The pathophysiology of proposed mechanisms of this is poorly understood. However, this review will examine the current literature and evidence for Vitamin D’s role in IBD.

## 2. Methods

A literature search was performed on PubMed. We used the search terms “Crohn’s Disease”, “Ulcerative Colitis”, “Inflammatory Bowel Disease” and “Vitamin D”. We also reviewed the references of retrieved articles and categorized them under different sections of this review.

## 3. Vitamin D Synthesis and Metabolism

Vitamin D is fat soluble and is absorbed in the small intestine with dietary fat. It exists in two major forms, vitamin D2 or ergocalciferol (from plants, yeast and fungi) and vitamin D3 or cholecalciferol (from oily fish and egg yolk). Vitamin D3 is the most physiologically relevant form of vitamin D and is synthesised endogenously in the skin upon exposure from UV light (wavelengths of 270–300 nm).

Orally ingested vitamin D is absorption into the intestines, it is incorporated into chylomicrons and transported in the venous circulation by vitamin D binding proteins and enters the liver. It is here that it is converted to 25(OH)D3. It is then finally metabolised in the kidneys into 1,25(OH)_2_VitD, which is the most physiologically active metabolite [6].

Vitamin D is also be metabolised by cells of the immune system. Therefore 1,25(OH)_2_VitD can be locally concentrated in lymphoid micro-environments, potentially increasing undesirable side effects, such as hypercalcaemia and bone resorption. Activated T-cells can perform the final step of converting 25(OH)VitD to 1,25(OH)_2_VitD. Finally the enzyme 24-hydroxylase catabolises 1,25(OH)_2_VitD to its inactive metabolite (calcitroic acid), where it is then excreted in the bile [7,8,9,10].

## 4. Epidemiology

IBD patients are at risk of a number of vitamin and mineral deficiencies. A study by Alkhouri *et al.* (2006) looked at 61 IBD patients and compared vitamin and zinc deficiency prevalence with 61 age and sex matched controls. They found that both IBD patients and controls had a high prevalence of Vitamin D deficiency (62% *vs.* 75%), thus showing no correspondence with disease in this study [11]. There are even a number of studies that show vitamin D levels can be low or normal in IBD patients, and that also vitamin D deficiency may be as a consequence of the disease itself. There are several confounders regarding vitamin D status of IBD patients. Firstly, patients with IBD may tend to spend more time indoors due to exacerbations. Secondly, patients with CD may have malabsorption of vitamin D and lastly, it has been shown that inflamed tissue expresses CYP24A1 and CYP27B1, which results in the consumption of 25(OH)VitD [12]. Thirdly, patients may stay indoors due to photosensitivity after starting medications, such as Mesalazine [13], or to decrease the risk of skin cancer from Azathioprine, whereby patients are instructed to use sun protection [14].

Some have also argued that IBD patients may be deficient in vitamin D due to steroid usage [4]. However, a study by Lamb *et al.* (2002) found bone mineral density was reduced at diagnosis, before steroids were initiated [15].

Perhaps there are other factors determining vitamin D status. Gilman *et al.* (2013) looked at Irish CD patients in a cross-sectional observational study. With multiple regression analysis, they showed that summer vitamin D levels were positively associated with use of vitamin D supplements (*p* = 0.033) and negatively associated with smoking (*p* = 0.006) and being male (*p* = 0.063). In winter, use of vitamin D supplements and (*p* = 0.041) and sun habits (*p* = 0.066) were positively associated with vitamin D levels, whilst small intestine involvement (*p* = 0.005) and Body Mass Index (BMI) (*p* = 0.083) were negatively associated. They found no associations with age, dietary intake of calcium or vitamin D, steroid use or resection [16]. Suibhne *et al.* (2012) also found vitamin D deficiency was higher in the winter than summer (68% *vs.* 50%; *p* < 0.0001), remained high in the summer (50%) and was associated with smoking [17].

Further to this, it has been reported that latitude may affect IBD prevalence in Europe [18,19,20]. Khalili *et al.* (2012) reported that in a group of women in North America, increasing latitude was associated with an increased incidence of CD and UC [21]. Nerich *et al.*’s (2011) geographic study reported that those with low sunlight exposure may be associated with an increased incidence of CD, but there was no association between sunlight and UC [22].

However, a similar study in Japan by Tajika *et al.* (2004) found that although CD patients did not have lower vitamin D levels than controls, vitamin D levels were related to disease duration (r = 0.46, *p =* 0.003), Crohn’s Disease Activity Index (CDAI) score (r = 0.44 *p =* 0.005), International Organisation for the Study of Inflammatory Bowel Disease score (r = 0.30 *p ≤* 0.05), serum value of ferritin (r = 0.34 *p =* 0.03), serum value of C-reactive protein (r = 0.34 *p =* 0.03), total cholesterol (r = 0.31 *p =* 0.03) and intact parathyroid hormone (r = 0.23 *p* < 0.05). They also found that duration of disease and CDAI score could predict the occurrence of vitamin D deficiency (*p =* 0.0004) and thus suggest that vitamin D deficiency should be tested in patients with disease for greater than 15 years [23].

Ethnicity may also affect vitamin D levels in IBD patients. Fu *et al.* (2012) found South Asians had decreased vitamin D levels. (All South Asians *vs.* all Caucasians in study—58.6% *vs.* 30.8%, *p* = 0.01; South Asians with CD *vs.* Caucasians with CD—85.7% *vs.* 32.3%, *p* = 0.01) [24]. Chatu *et al.* (2013) looked at factors associated with vitamin D deficiency in a multicultural cohort and found a difference in the vitamin D level between non-Caucasians (Asian and Black) and Caucasians (28 nmol/L (IQR 17–41) *vs.* 41nmol/L (IQR 25-63), *p* < 0.0001), with severe deficiency more prevalent in non‑Caucasians compared with Caucasians (44% *vs.* 24%, *p* < 0.05) [25].

A study by Veit *et al.* (2014) looked at levels of serum 25(OH)VitD in children and adolescents with IBD, and found no difference, although, they did find that those with active IBD and a high ESR had significantly lower levels of vitamin D (*p*=0.025), as well as controls (65.3 ± 28.0 nmol/L *vs.* 49.5 ± 25.23, *p* = 0.045), thus suggesting that patients with a high ESR should be monitored for vitamin D deficiency [26].

## 5. Vitamin D and Bone Health in IBD

Comparing UC and CD, Ardizzone *et al.* (2000) compared bone metabolism and turnover in both diseases. They found only 8% of CD patients and 15% UC patients had a normal bone mineral density, with 55% (CD) and 67% (UC) were osteopenic, and 37% (CD) and 18% (UC) were osteoporotic. They also found that bone turnover was higher in UC patients than CD patients (increased levels of biochemical markers of bone turnover including osteocalcin and type 1 collagen C-terminal peptide). Osteopenia was related to disease duration in CD, but in UC osteopenia was associated with glucocorticoid treatment and the male sex [27].

Correlation has also been found with time of disease remission and bone density. Reffitt *et al.* (2003) found that, after evaluating 137 patients (64 UC and 73 CD), patients with disease in remission for more than three years had a normal mean Z-score (bone mass density relative to the age-standardised mean), which was significantly higher than those with active disease. They also interestingly found that those on azathioprine and in remission had higher mean Z-scores when compared to those with active disease and to those who were not taking azathioprine [28].

Schoon *et al.* (2001) looked at bone turnover in those with longstanding CD patients in remission. They analyzed markers of bone formation, such as osteocalcin and bone-specific alkaline phosphatase. These were lower in CD patients when compared to controls (osteocalcin: *p* = 0.027, bone-specific alkaline phosphatase: *p* < 0.001) [29].

## 6. Genetics

The vitamin D receptor gene represents a strong candidate gene for susceptibility to IBD, as it lies within a region of Chromosome 12, which has already been linked to IBD [30,31]. There are a number of studies that suggest a genetic influence on vitamin D levels.

Xue *et al.* (2013) performed a meta-analyses looking at vitamin D Receptor (VDR) polymorphisms (Taql, Bsml, Fokl, and Apal). This revealed that in Asians, the ff genotype of Fokl was associated with an increased risk of UC (OR = 1.65; 95% CI, 1.11–2.45) with “a” allele carriers of the Apal polymorphism having a protective factor against developing CD (OR = 0.81; 95% CI, 0.67–0.97). The Taql tt genotype increased the risk of CD in Europeans, (OR = 1.23; 95% CI, 1.02–1.49) and in European males a moderately elevated risk of UC (OR = 1.56; 95% CI, 1.02–2.39) and CD (OR = 1.84; 95% CI, 1.19–2.83) [32].

Simmons *et al.* (2000) looked at 158 UC patients, 245 CD patients and 164 cadaveric renal allograft donor controls and found a higher frequency of the tt genotype of the Taql polymorphism amongst CD patients (frequency 0.22) when compared to UC patients (0.12) or controls (0.12) (odds ratio 1.99; 95% confidence interval [CI] 1.14–3.47; *p* = 0.017) [30].

The Bsml VDR polymorphism was analysed in an Ashkenazi Jewish population, and found that the frequency of the BB genotype was higher in Ashkenazi UC patients compared with Ashkenazi controls (0.21 *vs.* 0.11 *p* = 0.042, odds ratio 2.27, (95% confidence interval [CI] 1.06–4.9) [33]. In an Iranian cohort of CD patients, Naderi *et al.* (2008) found a probable association with the ff genotype of the Fokl polymorphism (*p* < 0.001) [34].

Eloranta *et al.* (2008) added to this body of work by looking at two single nucleotide polymorphisms of Vitamin D Binding protein (DBP) and found the DBP 420 variant Lys was less frequent in IBD cases compared with controls (allele frequencies, *p* = 0.034; homozygous carrier genotype frequencies, *p* = 0.006). Another DBP polymorphism (DBP 416) was not associated with IBD, but the haplotype consisting of 416 Asp and 420 Lys was more frequent in controls, especially when compared to UC patients (Odds ratio, 4.390) [35].

Thus, there are suggestions that genetics does have an influence on vitamin D levels. What is unclear is how these genes are related to disease aetiopathogenesis in IBD and this needs further elucidation in future studies.

## 7. The Role of Vitamin D in Immunity

The emergence of vitamin D as an immune regulator has prompted investigation of its role in the adaptive and innate immune system. One study suggested that some cases of Delayed Hypersensitivity may be related to vitamin D deficiency [36]. It is also known that dendritic cells express receptors for 1,25(OH)_2_VitD, which may indicate an activating, inhibiting or modifying effect on dendritic cell function [37]. There is also a suggestion that, in a murine model, vitamin D can induce tolerance to fully mismatched islet murine allografts by enhancing regulatory T cells to mediate transplantation tolerance [38].

Zhang *et al.* (2012) unveiled a potential mechanism for this immunomodulation. They investigated the inhibitory effects of vitamin D on Lipopolysaccharide (LPS)-stimulated inflammatory response in human monocytes and found that both 1,25(OH)_2_VitD3 and 1,25(OH)_2_VitD3 dose dependently inhibited LPS-induced p38 phosphorylation at physiological concentrations, and inhibited Interleukin‑6 (IL-6) and Tumour Necrosis factor-α (TNF-α) production by human monocytes. They also found that after starting vitamin D treatment, MAPK Phosphatase-1 (MKP-1) was significantly upregulated in human monocytes. In Bone Marrow-derived macrophages from a murine sample, those samples that were MKP1(−/−), inhibition of LPS-induced p38 phosphorylation was completely abolished and, thus, LPS-induced IL-6 and TNF-α release was also reduced. Thus they concluded that upregulation of MKP-1 allows for vitamin D inhibition of LPS-induced p38 activation and thus cytokine production in monocytes and macrophages [39].

Liu *et al.* (2009) reported that synergy between IL-1β and Vitamin D activation was necessary for the activation of the TLR (Toll-like Receptor) induced antimicrobial response against intracellular pathogens [40]. Vitamin D may also trigger autophagic death on human myeloid leukaemia cells [41].

## 8. Vitamin D and Antimicrobial Peptides

Vitamin D has been implicated as an immunomodulatory molecule for its involvement in the immune response against *M. tuberculosis*, involving the recognition of bacterial lipoproteins by TLRs, induction of CYP27B1, which converts 25(OH)VitD to 1,25(OH)_2_VitD and upregulates and activates the Vitamin D receptor (VDR), and it has long been known that activation of VDRs in monocytes cause an anti-microbial response against *M. tuberculosis*. Vitamin D Response Elements (VDRE) have been shown to regulate the antimicrobial peptides cathelicidin and Defensin Beta-4 (DBEF-4) and that vitamin D induced expression of cathelicidin was necessary for an antimicrobial response against *M. tuberculosis* [42,43,44,45,46,47,48,49].

The relationship of vitamin D and cathelicidin was also confirmed by Adams *et al.* (2009). They assessed 50 patients attending a bone clinic and looked at cathelicidin antimicrobial peptide (hCAP) *in vivo* and *ex vivo*. In those who were vitamin D insufficient, monocytes showed an increased expression of the vitamin D activating enzyme (CYP27b1) but a decreased expression of hCAP mRNA. After receiving treatment, expression of hCAP correlated with 25(OH)VitD (r = 0.649, *p* < 0.001), suggesting that vitamin D has a key role in maintaining localised production of antimicrobial hCAP following TLR activation [50].

Liu *et al.* (2006) found that TLR activation of human macrophages upregulated the expression of VDR and 1α-hydroxylase genes, leading to the induction of cathelicidin and, thus, killing of *M. tuberculosis*. In African-American individuals, it is known that they have low 25-hydroxyvitamin D and are also inefficient in inducing cathelicidin mRNA, thus supporting a link between vitamin D and susceptibility to microbial infection [45].

## 9. Th2 Cell Development

Boonstra *et al.* (2001) demonstrated that 1,25(OH)_2_VitD drives Th2 cell development. They observed the effects of vitamin D on CD4 cells derived from mice and found that vitamin D affected Th cell polarisation by inhibiting Th1 (Interferon-γ production) with an increased expression of Th2 transcription factors (GATA-3 and c-maf) after vitamin D3 treatment. Thus, this study suggests that vitamin D may be useful in the treatment of Th1 driven autoimmune disease [51].

## 10. B Cell Function

Vitamin D may also be involved in B cell function. The effects of 1,25(OH)_2_VitD on B cell responses was analysed by Chen *et al.* (2007), which showed that the vitamin inhibited the ongoing proliferation of B cells, and induced their apoptosis, whilst also having an effect on generation of plasma cells and postswitch memory B cells [9].

## 11. Murine Models

Froicu *et al.* (2006) looked at IL-10 knockout mice as well as VDR knockout mice to determine the effect of the VDR on immune function and inflammation. Those with both IL-10 and VDR knockouts developed severe IBD involving all areas of the small intestine and colon. Those with just IL-10 knockout developed a less severe IBD, suggesting VDR deficiency exacerbates IBD severity [52].

Daniel *et al.* (2007) induced colitis in mice using Trinitrobenzene sulfonic acid and analysed colonic tissue microscopically and macroscopically, and found that Calcitriol promoted regulatory T cell profiles, and an increase in TGF-β, IL-10, FoxP3 and CTLA-4 [53].

Lagishetty *et al.* (2010) performed a similar study on mice, and used dextran sodium sulphate (DSS) to induce colitis. Those mice that were on a vitamin D deficient diet showed greater DSS-induced weight loss (9% *vs.* 5%), increased colitis (4.71 ± 0.85 *vs.* 1.57 ± 0.18), and splenomegaly relative to mice on a normal diet. Vitamin D deficient mice also had a 50-fold increase in the amount of gut bacteria, thus suggesting a role for vitamin D in homeostasis of gut bacteria and immune regulation [54]. Kong *et al.* (2008) also looked at DSS-induced colitis in mice, with comparison of histology between VDR (−/−) and VDR (+/+), and found VDR (−/−) were more susceptible to mucosal injury, thus suggesting vitamin D has an important role in mucosal barrier homeostasis [55].

Liu *et al.* (2008) looked at the enzyme Cyp27b1 (CYP27b1 in humans), which is known to be upregulated in Crohn’s disease and catalyses the endocrine synthesis of 1,25(OH)_2_D in the kidney. It is also present in extra-renal sites and in the colon. Liu *et al.* demonstrated that mice with DSS-induced colitis showed a decreased expression of Cyp27b1 in the kidneys, but an increased expression in the proximal colon compared with controls (*p* < 0.001). Those mice that were Cyp27b1 (−/−) showed decreased IL-10 in the proximal colon and Toll-like receptors 2 and 4 in the distal colon as well as decreased levels of circulating 1,25(OH)_2_VitD, thus implicating vitamin D affecting colitis in DSS‑treated mice [56].

There have also been studies looking at the effects of vitamin D analogues on DSS-induced colitis in mice. The analogue TX527 seemed to significantly decrease disease scores by suppressing bleeding and diarrhoea, and histologically IL-1, IL-6, IFN-γ and TNF-α were downregulated in colonic mucosa and, thus, may be of therapeutic value in IBD [57].

Another study performed microarray analysis of colons of vitamin D treated mice who have experimentally induced IBD, and found 239 genes inhibited, which interestingly included 3 TNF-α related genes (TNF-α, TNF receptor and lipopolysaccharide-induced TNF-α factor), suggesting vitamin D’s role as an immunodulator even further [58].

Further to this, Zhu *et al.* (2005) looked at IL-10 knockout mice, which are known to develop colitis. Mice fed either calcium or 1,25(OH)_2_VitD developed intermediate IBD, but those fed both had the mildest form of IBD and the lowest concentrations of TNF-α. They also found that LPS-induced TNF-α production was inhibited by vitamin D administration and was associated with decreased colitis severity [59].

## 12. Disease Modification and Vitamin D

Ham *et al.* (2014) looked at prospectively collected vitamin D samples of CD patients. Those with active CD had lower vitamin D levels than those patients in remission (independent of season or use of vitamin D supplementation). After starting infliximab, significant increases in vitamin D levels were observed, suggesting circulating vitamin D levels are influenced by disease activity [60]. However, in another study by Grunbaum *et al.* (2013), those with mild or inactive disease had similar levels of vitamin D to controls [61].

Leichtman *et al.* (1991) compared vitamin D levels of patients with small (<100 cm), intermediate (100–300 cm) and large bowel resections (>300 cm) and found that intestinal absorption of both cholecalciferol and 25-hydroxycholecalciferol are reduced in CD patients, with the degree of deficiency related to extent of bowel resection [62]. However, Vogelsang *et al.* (1997) performed a study measuring vitamin D levels after oral consumption and found no correlation with previous resections and vitamin D malabsorption in CD patients [63].

Jorgensen *et al.* (2013) looked at 182 CD patients and 62 controls, and found associations of low 25(OH)VitD with active Crohn’s disease. Patients who took oral vitamin D supplementation had a lower CD activity index (*p* < 0.05), but overall, Crohn’s disease patients, intriguingly, did not have different vitamin D levels compared with healthy controls [64].

Hlavaty *et al.* (2014) found that serum vitamin D concentration correlated with health related quality of life, but this was found to only be significant in the Winter/Spring period (assessed using the short health related IBD questionnaire, *p* = 0.04) [5].

A pilot study was performed in 18 patients with mild-moderate CD to determine the dose of vitamin D to raise serum levels to above 40 ng/mL. They found that when vitamin D was increased, mean CDAI scores reduced from 230 ± 74 to 118 ± 66 (*p* < 0.0001), and quality of life scores also improved (*p* = 0.0004), although this was a small study [65].

A systematic review by Nicholson *et al.* (2012) found that all four studies in the review showed improvement in disease activity with vitamin D supplementation, but they also suggest that large, high quality placebo-controlled randomized controlled trials are needed to fully explore the benefits of vitamin D supplementation in IBD [66].

## 13. Differences between CD and UC

Abreu *et al.* (2004) demonstrated that a subset of Crohn’s patients had a higher level of circulating 1,25(OH)_2_VitD when compared to UC (57 pg/mL) *vs.* UC (41 pg/mL) (*p* = 0.0001) as well as immunohistochemistry and RT-PCR showing an increased level of 1-α hydroxylase in CD [67].

A prospective cohort study by Ananthakrishnan *et al.* (2012) looked at the risk of developing CD and UC in 72,719 women enrolled in a Nurses’ Health Study and found that those with the highest predicted plasma level of Vitamin D had a significantly reduced incidence of CD when compared to those in the lowest quartile (40% reduced risk), but no reduced risk of UC [68].

## 14. Conclusions

An increasing number of epidemiological, genetic, basic science and animal model studies support the concept that vitamin D regulation may partly determine occurrence and course of IBD, which warrants further study. Clinical studies have confirmed that vitamin D deficiency is common in this patient group. Data is starting to emerge that identifying those with a deficiency and correcting may improve health outcome measures.

There remain unanswered questions. It is yet unclear as to whether vitamin D deficiency is a causative factor for IBD or a risk factor and there is widespread variability of results amongst IBD patients. Thus more robust studies are required to elucidate whether there is a role for vitamin D in the management of IBD patients.

There also remain questions as to whether vitamin D modifies levels of inflammation, or its effect on disease severity. It is also not known whether vitamin D deficiency is associated with a clinical phenotype or its influence on risk of colorectal cancer in IBD patients [69].

It is clear that further studies are necessary to fully evaluate the role of vitamin D in IBD. Until the exact role of vitamin D in IBD is established it would seem sensible to identify and treat any IBD patients with a vitamin D deficiency.
